# Rhombencephalosynapsis With Obstructive Hydrocephalus: A Rare Presentation of the Cerebellar Anomaly on MRI Findings

**DOI:** 10.7759/cureus.39969

**Published:** 2023-06-05

**Authors:** Shreya Khandelwal, Rajasbala Dhande, Harshith Gowda, Gaurav Mishra, Ramesh Khandelwal

**Affiliations:** 1 Radiodiagnosis, Jawaharlal Nehru Medical College, Datta Meghe Institute of Higher Education and Research, Wardha, IND; 2 Radiodiagnosis, Khandelwal Diagnostic Center, Dhamtri, IND

**Keywords:** corpus callosum, rhombencephalosynapsis, mri, hydrocephalus, cerebellar abnormalities

## Abstract

Rhombencephalosynapsis is an exceptionally uncommon cerebellar anomaly characterized by the absence or underdevelopment of vermal axons, the presence of dentate nuclei, and the fusion of cerebral hemispheres. Depending on the presence or absence of additional supratentorial anomalies, the prognosis and clinical appearance can vary widely. Here, we present the case of a consanguineous-parent newborn boy, aged four days, who was diagnosed with the use of an MRI. The child had spastic diplegia, bone anomalies, and facial dysmorphism. Slight hydrocephalus, hypogenesis of the corpus callosum, and agenesis of the septum pellucidum were some of the additional supratentorial abnormalities. This study details the clinical and MRI findings, as well as a possible etiology, of this illness.

## Introduction

Rhombencephalosynapsis, a rare genetic disorder, is defined by the connection of the superior cerebellar peduncles, dentate nuclei, and cerebellar hemispheres and the lack of the cerebellar vermis, which results in a distinctive keyhole shape in the fourth ventricle [1]. On posterior coronal imaging, the cerebellar hemispheres appear to be connected in a horizontal foliate pattern. A dorsoventral patterning deficiency in the rostral dorsal midline sections of the rhombomere [1], which are situated just beneath the isthmus and surround the upper fourth ventricle, is most likely the cause of rhombencephalosynapsis, despite the genetic etiology of the condition still being unclear [2,3]. Though the majority of patients lack a syndromic profile, they do exhibit evidence of delayed motor development, aberrant eye movements, and stereotypical head movements [4]. The long-term state of cognitive function can range greatly, from severe impairment to normal. Rhombencephalosynapsis is a characteristic of Gómez-López-Hernández syndrome in some individuals (parietal alopecia, trigeminal anesthesia, and craniofacial dysmorphic signs) [5]. 

In 1916, Obersteiner first recognized rhombencephalosynapsis in a clerical worker who had committed suicide at the age of 28 [6]. Magnetic resonance imaging has come a long way since its infancy, and the growing expertise of pediatric neurologists and neuroradiologists has led to an increase in the detection of rhombencephalosynapsis both during and after pregnancy [7,8]. There is a continuum of severity of rhombencephalosynapsis, from severe cases where the complete vermis is absent (including the nodulus) to mild cases where only the central portion of the vermis has fused, typically the posterior vermis, with portions of the anterior vermis visible beyond the fusion [1]. The loss of the posterior vermis and nodulus with the presence of some remnant anterior vermis characterizes an unusual partial rhombencephalosynapsis, which occurs in a limited number of cases [1].

## Case presentation

A healthy baby boy was delivered vaginally at term to G3P1L1A1. No serious medical conditions, such as congenital abnormalities, were present in the family tree. The birth weight was 2.7 kg. On day four post-birth, facial dysmorphia was found after a thorough checkup (low-set ears and hypertelorism). The neurological evaluation revealed lower limb hyperreflexia, spastic diplegia, and bilateral Babinski's sign. On closer inspection, we noticed scoliosis and genu valgum. Spinal radiographs exposed segmentation anomalies in the thoracic spine at vertebrae T8 and T9, along with mild scoliosis. 

**Figure 1:**
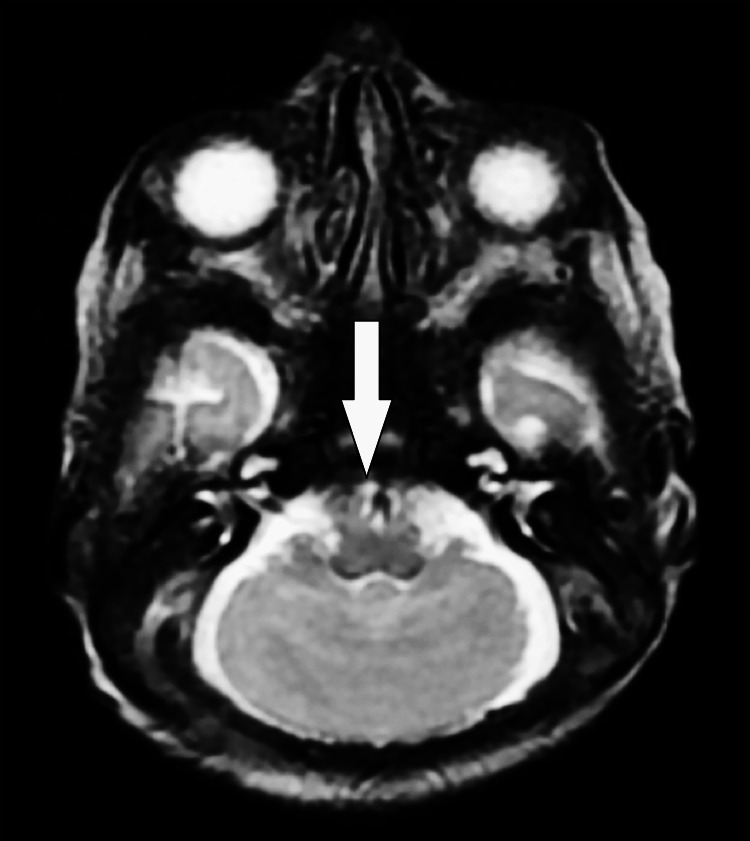
Axial view MRI T2WI axial view shows fused cerebellar hemispheres across the midline with the absence of vermis highlighted by the 'Donald Duck' sign with dilated lateral and third ventricle secondary to aqueductal stenosis T2WI: T2 weighted image

**Figure 2:**
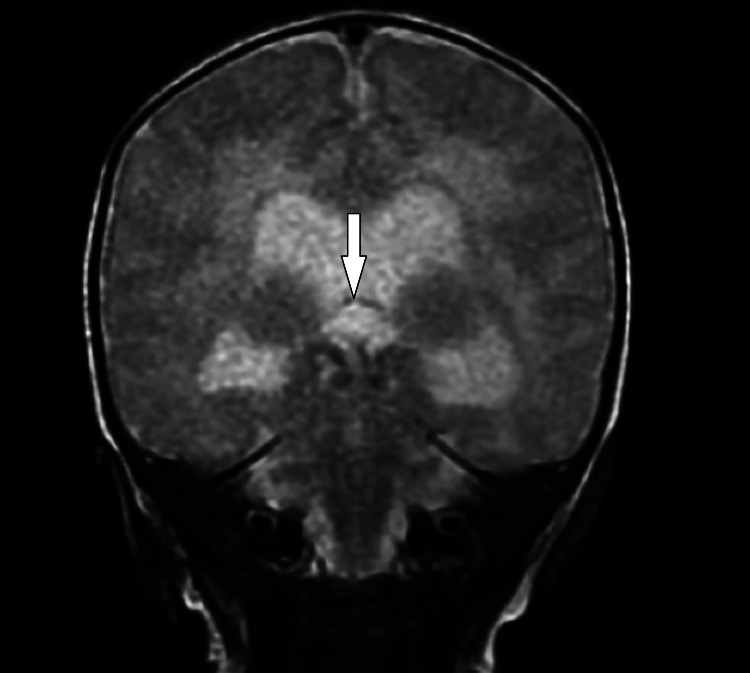
Coronal view MRI T2WI coronal view shows fused cerebellar hemispheres across the midline with the absence of vermis accentuated by the 'Donald Duck' sign with dilated lateral and third ventricle secondary to aqueductal stenosis T2WI: T2 weighted image

**Figure 3:**
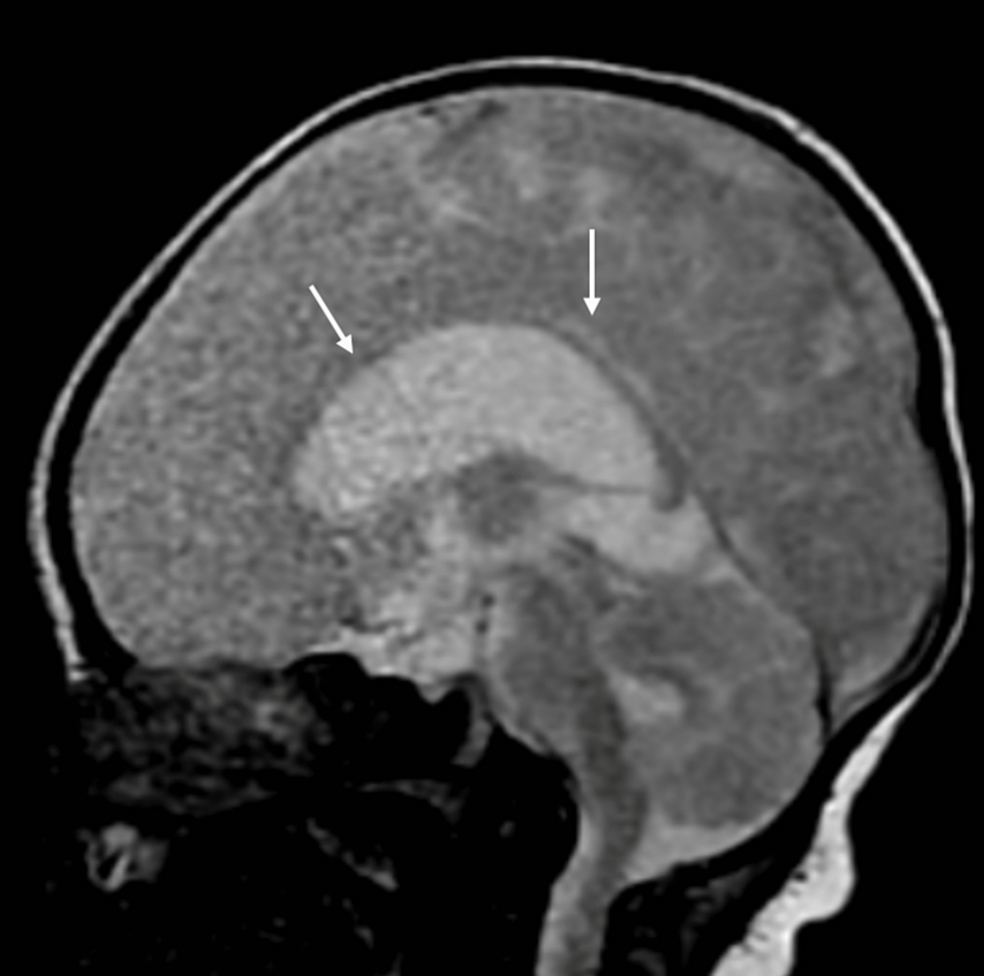
Sagittal view MRI T2WI sagittal view shows hypogenesis of the corpus callosum T2WI: T2 weighted image

A thorough ophthalmic evaluation showed healthy retinas and transparent corneas. Among the many anomalies seen by the cranial MRI were hypogenesis of the corpus callosum, biventricular hydrocephalus, full agenesis of the vermis and the agenesis of the septum pellucidum, dentate nuclei, fusion of the cerebellar hemispheres, and superior cerebellar peduncles. The optic chiasm and pituitary gland were both in good condition as shown in Figures 1-3. No further CNS or extra-CNS abnormalities were discovered, and chromosomal analysis showed a normal karyotype of 46, XY.

## Discussion

An MRI can currently be used to diagnose this abnormality in utero, and in certain cases, this has been done [7]. A total of 50 instances of rhombencephalosynapsis have been reported to date, with an MRI diagnosis made in about 36 cases. Two pediatric patients with normal growth of the anterior vermis and nodulus and a deficiency in part of the posterior vermis were recently identified as having partial rhombencephalosynapsis [9,10]. These findings imply that rhombencephalosynapsis needs to be seen as a deformity with a range of severity. Although there are occasional reported cases of adult patients in the literature, juvenile patients are the focus of the majority of reports [11,12]. Sener reported that among 3000 pediatric patients who had head MRIs, the frequency of rhombencephalosynapsis was 0.13% [13]. Most of the cases that have been published have been sporadic. Although chromosomal analysis in our instance was normal and there was no family history of deformity, paternal consanguinity may point to an autosomal recessive inheritance. But in India, consanguinity is a typical constellation. Parents in two further examples recorded in the literature share a common constellation of consanguinity and come from an ethnic background [14]. An autosomal recessive inheritance appears highly improbable because no occurrence in siblings has ever been noted.

With its excellent resolution and multiplanar imaging, MRI is the go-to diagnostic tool for evaluating cerebellar disorders. The MRI imaging in our case revealed the superior cerebellar peduncles, dentate nuclei, and cerebellar hemispheres that had fused. Images taken near the crown revealed a horizontal arrangement of leaves with no visible vermis [15,16]. Developmental abnormalities in the cerebellum between 28 and 41 days of gestation result in rhombencephalosynapsis. Inappropriate abnormalities in the supratentorial region of the brain are common at this point in gestation. It is unclear what causes this midline deformity and what role pathophysiology plays in its development. The standard explanation for rhombencephalosynapsis was to attribute it to an abnormal progression of the vermis, followed by a union of the cerebellar hemispheres.

## Conclusions

In conclusion, the clinical manifestation of rhombencephalosynapsis varies widely; spastic diplegia in children is one example of this anomaly. Compared to extracranial anomalies, supratentorial causes of rhombencephalosynapsis in children are more common. Despite progress in understanding what causes rhombencephalosynapsis, genetic defects remain a potential problem. More research and speculation regarding possible faulty genes may shed light on the pathophysiology of this unusual cerebellar disorder.
